# High Thermoelectric Performance of Cu-Doped PbSe-PbS System Enabled by High-Throughput Experimental Screening

**DOI:** 10.34133/2020/1736798

**Published:** 2020-03-07

**Authors:** Li You, Zhili Li, Quanying Ma, Shiyang He, Qidong Zhang, Feng Wang, Guoqiang Wu, Qingyi Li, Pengfei Luo, Jiye Zhang, Jun Luo

**Affiliations:** ^1^School of Materials Science and Engineering, Shanghai University, 99 Shangda Road, Shanghai 200444, China; ^2^Materials Genome Institute, Shanghai University, 99 Shangda Road, Shanghai 200444, China

## Abstract

Recent advances in high-throughput (HTP) computational power and machine learning have led to great achievements in exploration of new thermoelectric materials. However, experimental discovery and optimization of thermoelectric materials have long relied on the traditional Edisonian trial and error approach. Herein, we demonstrate that ultrahigh thermoelectric performance in a Cu-doped PbSe-PbS system can be realized by HTP experimental screening and precise property modulation. Combining the HTP experimental technique with transport model analysis, an optimal Se/S ratio showing high thermoelectric performance has been efficiently screened out. Subsequently, based on the screened Se/S ratio, the doping content of Cu has been subtly adjusted to reach the optimum carrier concentration. As a result, an outstanding peak zT~1.6 is achieved at 873 K for a 1.8 at% Cu-doped PbSe_0.6_S_0.4_ sample, which is the superior value among the *n*-type Te-free lead chalcogenides. We anticipate that current work will stimulate large-scale unitization of the HTP experimental technique in the thermoelectric field, which can greatly accelerate the research and development of new high-performance thermoelectric materials.

## 1. Introduction

In 2011, the Obama administration launched the “Materials Genome Initiative” (MGI) project, which was aimed at reducing the cost and shortening the research and development cycle for exploring new materials [1]. By virtue of the combined high-throughput (HTP) computational and experimental techniques, the discovery, optimization, and deployment of new materials will be greatly accelerated [1]. Recent advances in computational power and machine learning have led to great achievements in theoretical prediction of new functional materials in the fields of catalysis [2], lithium battery [3], photovoltaics [4], and thermoelectrics [5–9]. In particular, Google recently announced the realization of quantum supremacy by using a programmable superconducting processor, indicating great advancement in developing next-generation computers [10]. It is reasonable to believe that in the foreseeable future, the ability of HTP computational power will be boosted by using the next-generation supercomputer. However, the development of the HTP experimental technique is relatively slow, which seriously lags behind that of the theoretical prediction. Historically, discovery and optimization of functional materials have long depended on the traditional Edisonian trial and error approach, which leads to costly and time-consuming procedures in verifying the massive theoretical HTP results [11]. Therefore, it is of great theoretical and practical significance to exploit experimental HTP techniques, which may bring about revolutionary breakthrough in material research.

Optimizing the performance of a thermoelectric material is relatively difficult due to its adversely interdependent transport parameters [12–15]. Specifically, the performance of a thermoelectric material depends on the dimensionless figure of merit, zT, which is defined as zT = *σS*^2^*T*/(*κ*_e_ + *κ*_L_), where *σ*, *S*, *κ*_e_, *κ*_L_, and *T* are the electrical conductivity, Seebeck coefficient, electrical thermal conductivity, lattice thermal conductivity, and absolute temperature, respectively [16]. Thus, in order to improve thermoelectric performance, these transport parameters should be synergistically optimized. Conventionally, doping or alloying at specific lattice sites may impose great influence on both the electronic and phonon transport properties of a thermoelectric material. Up to now, the multidoping (alloying) strategy has been demonstrated to be a highly effective approach in boosting the performance of the state-of-art bulk thermoelectric materials. For example, in order to obtain ultrahigh zT values, doping or alloying with at least two elements is necessary for the GeTe- [17, 18], SnTe- [19, 20], and PbTe- [21–23] based compounds. Apparently, it becomes increasingly difficult to achieve satisfactory compositions for the above-mentioned system via serial experimental synthesis and characterization processes. Therefore, it should be more favorable to employ an experimental HTP technique to screen out an appropriate target composition instead of the trial and error approach before concentrating on subtle experimental optimization.

Generally, the HTP sample fabrication technique can be divided into two categories, i.e., material gene chip technology and continuously gradient composition preparation technique. The latter is usually adopted to fabricate bulk functional gradient materials (FGM) for transport property distribution characterization in thermoelectric communities due to its favorable composition features [24]. Januszko et al. prepared gradient samples using sedimentation of atoms under a strong gravitational field and obtained a large diffusion area in a Bi_1‐*x*_Sb_*x*_ alloy [25]. Hedegaard et al. prepared the (PbTe)_1‐*x*_(SnTe)_*x*_ [26] and Ge_1‐*x*_Si_*x*_ [27] gradient samples by the Bridgman and Czochralski crystal growth method and used the Potential-Seebeck Microprobe (PSM) to investigate the electrical-transport properties. By the Bridgman method, Kohri et al. grew a Ge-graded (PbTe)_1‐*x*_(GeTe)_*x*_ sample and investigated the influence of the Ge content on the thermoelectric transport properties [28]. In addition to the Bridgman method, Gelbstein et al. prepared functionally graded (PbTe)_1‐*x*_(SnTe)_*x*_ samples using cold pressing followed by an annealing process and investigated the distribution of the Seebeck coefficients under different heat treatment conditions [29]. Recently, the same group paid attention to the influence of the PbI_2_ content on the thermoelectric properties of (Pb_0.95_Sn_0.05_Te)_0.92_(PbS)_0.08_ by a similar approach [30].

In our previous study, we found that dynamic doping effect of interstitial Cu was highly effective in enhancing the thermoelectric performance of *n*-type PbSe and PbTe [31, 32]. Herein, we move forward to the Cu-doped PbSe-PbS solid solution system and intend to realize perfect doping effect through manipulating the interstitial space. According to the phase diagram, PbSe and PbS form a complete solid solution in a wide temperature range [33]. S alloying is expected to enlarge the band gap and thus extends the working temperature range. Besides, by using more affordable elemental S, substantial reduction of the cost can be also achieved. Clearly, this system has two adjustable composition parameters, i.e., Cu content and the Se/S ratio. In order to obtain the optimized composition efficiently, we have therefore divided the experimental procedure into two steps. First, with a fixed Cu content of 2 at%, we have synthesized a bulk HTP sample with graded compositions through hot-pressing and subsequent annealing procedure. Then, by combining the HTP characterization technique with the theoretical analysis on the transport model, the best Se/S ratio was determined. Subsequently, based on the screened result, a more subtle experimental study was devoted to optimizing the carrier concentration through precise turning of Cu contents. As a result, an outstanding peak zT~1.6 at 873 K for a 1.8 at% Cu-doped PbSe_0.6_S_0.4_ sample is achieved, which is a superior value among the *n*-type Te-free lead chalcogenides.

## 2. Results and Discussion

**Figure 1:**
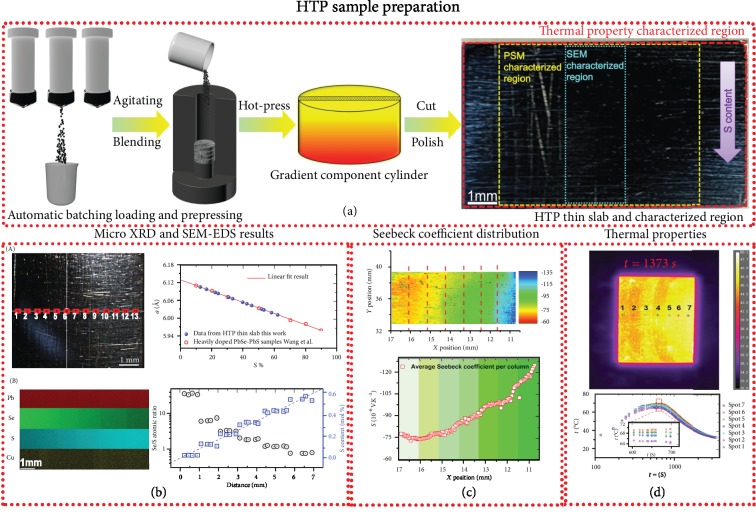
Experimental high-throughput screening strategy for the Cu-doped PbSe-PbS system. (a) HTP sample preparation process. (b) HTP structure and composition characterization. (c) Seebeck coefficient mapping of the HTP thin slab. (d) Thermal-transport property evaluation of the HTP thin slab. The red opened symbol presented in (b) was taken from Ref. [34]. The figures in (b) are the lattice parameters derived from micro XRD measurements at the labeled spots (A) and the SEM-EDS mapping results (B) for the HTP thin slab along the gradient direction. The data presented in (c) are the Seebeck coefficient distribution and the average value for each section (separated by red dotted lines). (d) Represents the upper surface temperature distribution and the time-dependent temperature variation of the marked spots for the HTP thin slab. The image of the HTP thin slab was captured by an infrared camera at *t* = 1373 s (the corresponding bottom temperature of the thin slab is ~250°C).

To screen out a satisfactory Se/S ratio efficiently, we have divided the HTP screening strategy into three steps: (1) HTP sample fabrication process, (2) HTP sample characterization, and (3) transport model analysis. The probably optimized Se/S ratio is determined through combining the results of step (2) and step (3) for further thermoelectric performance optimization. As shown in Figure 1(a), the HTP sample fabrication process consists of three procedures. First of all, two samples with nominal composition PbCu_0.02_S and PbCu_0.02_Se were synthesized by a traditional vacuum melting method followed by a very short period of ball-milling (5 min) to obtain precursor powders. Then, the precursor powders were weighed and loaded into a graphite die through our home-made automatic batching and prepressing system ([Supplementary-material supplementary-material-1]) according to a composition gradient of (1‐*x*)(PbCu_0.02_Se):*x*(PbCu_0.02_S) (*x* = 0.1, 0.2, ⋯, 0.7). The detail of the operating principle for the system can be found in our previous report [24]. After that, a high-density cylinder-like sample with composition gradient was obtained by induction hot-pressing and subsequent annealing process under vacuum. Finally, the HTP thin slab was carefully cut and polished for further characterization.

The crystal structure and composition distribution of the HTP thin slab was characterized and analyzed by microarea X-ray diffractometry and SEM-EDS. The results are presented in Figure 1(b). As shown in the upper left panel in Figure 1(b) and [Supplementary-material supplementary-material-1](a), the XRD patterns were collected from 13 micro regions labeled from 1 to 13. The diffraction patterns for all regions can be well indexed to the $\text{Fm}\bar{3}\text{m}$ space group and no obvious impurities are observed ([Supplementary-material supplementary-material-1]1(a)). Furthermore, it reveals that the diffraction peaks shift to higher angles from regions 1 to 13, indicating that the lattice parameter gradually decreases along the increasing direction of S contents ([Supplementary-material supplementary-material-1] and [Supplementary-material supplementary-material-1]), and thus, composition-graded PbSe-PbS solid solutions have been formed. As illustrated in the upper right panel in Figure 1(b), the actual composition for each region can be roughly obtained by Vegard's law, which is determined by the linear fitting results from literature [34]. The composition distribution of the HTP thin slab was further determined by the SEM-EDS analysis. It is worth noting that the area for SEM-EDS characterization is approximately close to 7 mm in length, which is too large that may result in a considerable error to determine the actual composition in a one-time measurement. Thus, we have divided this area into 14 small regions and then used EDS mapping to determine elemental distributions and compositions separately. The results are shown in the bottom panel of Figure 1(b). It can be seen that Pb and Cu elements are uniformly distributed, whereas Se and S exhibit a clear composition gradient along the entire length of the sample.

As shown in Figure 1(c), the Seebeck coefficient distribution of all the samples shows a clear layered feature with gradual change along the composition gradient. More precisely, with the decreasing Se/S ratio, the absolute value of the average Seebeck coefficient of each layer increases from 80 × 10^−6^ V·K^−1^ to 128 × 10^−6^ V·K^−1^ (see lower region in Figure 1(c)). According to our previous study on Cu-doped lead chalcogenides, the room-temperature solubilities of the interstitial Cu in PbSe and PbS were very small [31, 32], and both Cu-doped PbSe and PbS had similar carrier concentration at room temperature. This implies that the carrier concentration of each region of the HTP sample should be also similar at room temperature (this is further confirmed by our Hall effect measurement; see the carrier concentration and mobility in [Supplementary-material supplementary-material-1]). Therefore, the enhancement of the absolute value of the Seebeck coefficient with increasing S contents should be ascribed to the increased density-of-state effective mass near the conduction band edge (see the discussion below).

To screen out the favorable composition for high thermoelectric performance, both electrical and thermal properties should be measured. In the thermoelectric community, transport property measurement for the FGM was primarily focused on electrical properties, more precisely, the distribution of the Seebeck coefficient. To the best of our knowledge, HTP characterization of the thermal properties for the FGM sample remains scarce. Therefore, a home-made apparatus equipped with an *in situ* dynamic vacuum rapid-heating unit and an infrared camera with a 50 *μ*m micro lens was first developed and applied to characterize thermal properties of a sample with multiple composition areas. The images of the apparatus are presented in [Supplementary-material supplementary-material-1]. Apparently, at a given baseplate temperature, the surface temperature distribution of the sample should directly reflect the thermal conductivity of the corresponding area, enabling us to qualitatively estimate the thermal conductivity of each position.

Based on the apparatus, the thermal properties of the HTP thin slab were qualitatively evaluated under the dynamic vacuum atmosphere. The bottom surface of the HTP thin slab was quickly heated up to 250°C with a heating rate of 50°C/min, kept at this temperature for 400 s, and then cooled to room temperature by forced water cooling. The time-dependent temperature variation of several composition regions for the HTP thin slab and a photograph taken at *t* = 1370 s are presented in Figure 1(d). As shown in the upper panel in Figure 1(d), the surface temperature shows obvious difference along the gradient component, indicating the obvious discrepancy of the thermal conductivity among the corresponding areas. Specifically, spots 1, 2, and spot 4 (Figure 1(d)) show relatively low surface temperatures, suggesting lower thermal conductivities for such regions. This is further confirmed by the time-dependent surface temperature variation recorded by the infrared camera. As illustrated in Figure 1(d), spots 1, 2, and 4 show obvious lower surface temperatures and more gentle heating/cooling slopes, revealing lower thermal conductivities of these areas. The low thermal conductivities of spot 1 and spot 2 could be ascribed to the hierarchical phonon scattering for the compositions with low S alloying fraction, resulting in a dramatically decreased lattice thermal conductivity over a wide temperature range [31]. It is also found that spot 4 with the Se/S ratio approximately close to 1.5 also exhibits a lower thermal conductivity, which can be attributed to the alloying effect.

**Figure 2:**
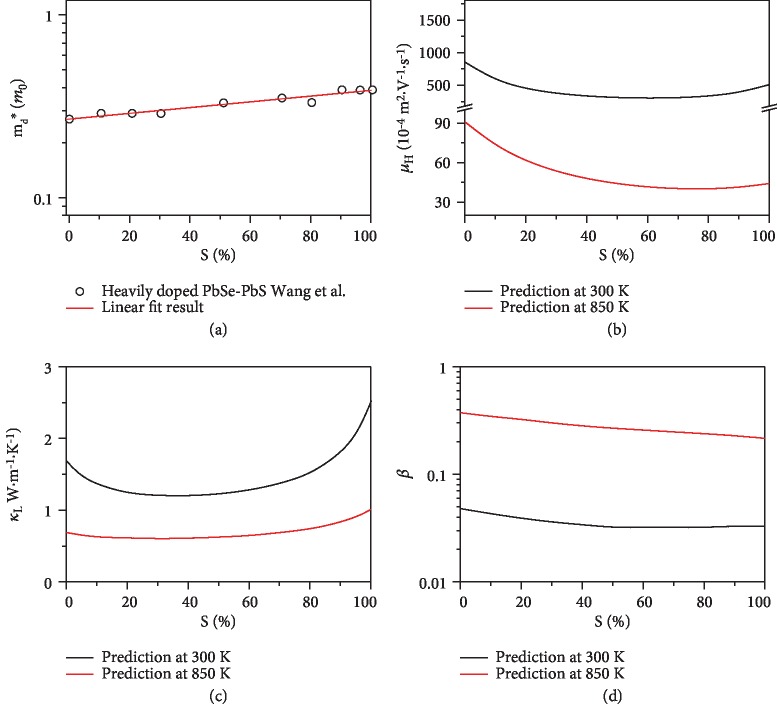
Influence of S alloying on physical properties of PbSe_1‐*x*_S_*x*_ predicted by theoretical considerations. S content dependence of (a) density-of-state effective mass of the conduction band, (b) Hall mobility, (c) lattice thermal conductivity, and (d) quality factor *β*. The opened symbol presented in (a) was taken from Ref. [34]. The solid lines in (b) are theoretical results of the Hall mobility based on the SKB model with the assumption that acoustic phonon and alloying scattering dominate electron transport. The Pisarenko relations for PbSe-PbS at 300 K and 850 K can be found in [Supplementary-material supplementary-material-1]. The solid lines in (c) are obtained from Klemens' model. The solid lines in (d) are the calculated results based on (a–c). The calculation detail and the physical parameters for modeling ([Supplementary-material supplementary-material-1]) can be found in the Supplementary material.

For the PbSe-PbS system, both the band gap (see [Supplementary-material supplementary-material-1]) and the density-of-state effective mass of conduction band (*m*_d_^∗^, see Figure 2(a)) increase upon S alloying, leading to increasing demand for optimal carrier concentration at higher temperature. According to our previous work, the space of the interstitial site is critical for a system to exhibit the dynamic doping effect. An “interstitial engineering” strategy has been thereby proposed, which is devoted to manipulating the dynamic doping effect [32]. In the S-alloyed PbSe system, due to the smaller atomic radius of the S atom, the tetrahedral interstitial space is noticeably increased upon S alloying, which may lead to a prominent dynamic doping effect at the high temperature range and provide an ideal platform for better utilization of the dynamic doping effect in a wide temperature range. In addition, the increased solubility of interstitial Cu owing to the enlarged interstitial space will also result in enhanced point-defect scattering and reduced lattice thermal conductivity. It can be expected that the peak zT might be greatly boosted in a Cu-doped PbSe-PbS solid solution through the prominent dynamic doping.

However, S alloying not only leads to prominent dynamic doping effect but also results in an alloying effect, which will simultaneously enhance the scattering of electrons and phonons in crystalline solids [35]. As shown in Figures 2(b) and 2(c), both the calculated Hall mobility and the lattice thermal conductivity show an asymmetrical variation with S content. As shown in Figure 2(b), the Hall mobility of the PbSe-PbS solid solution at 300 K drops dramatically as a small fraction of the S is alloyed (*x*~0.2) and then becomes approximately unchanged in the region of *x* = 0.2~0.8. A similar trend is also observed in the calculated lattice thermal conductivity. To theoretically evaluate the alloying effect on thermoelectric performance, the quality factor (*β*) has been hence calculated based on the results in thermal conductivity, Hall mobility, and *m*_d_^∗^. As shown in Figure 2(d), both at 300 K and 850 K, the quality factor decreases with increasing S content, indicating that the reduction of the lattice thermal conductivity cannot compensate for the deterioration in the Hall mobility. Therefore, it can be concluded that alloying effect is harmful to the overall thermoelectric performance of the PbSe-PbS system. All the calculation details can be found in the Supplementary material.

In order to balance the contributions of the dynamic doping effect and alloying effect, Se/S ratios must be carefully chosen for further performance optimization. Base on the HTP experimental results, the region near spot 4 has a moderate Seebeck coefficient and lower thermal conductivity, indicating that its related composition should have a suitable band gap, *m*_d_^∗^, and lattice thermal conductivity. Thus, the Se/S ratio~1.5 of spot 4 is screened out for further thermoelectric performance modulation.

**Figure 3:**
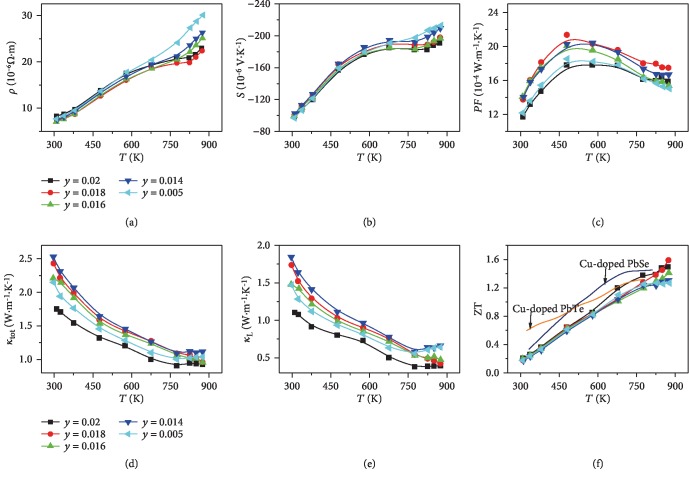
Temperature-dependent thermoelectric transport properties for PbSe_0.6_S_0.4_Cu_*y*_ (*y* = 0.005, 0.014, 0.016, 0.018, 0.02) samples: (a) Electrical resistivities; (b) Seebeck coefficients; (c) power factors; (d) total thermal conductivities; (e) lattice thermal conductivity; (f) Figure-of-merit zT. The solid lines in (f) are the temperature-dependent zT values for Cu-doped PbTe and PbSe with optimized Cu contents from Ref. [31, 32].

Subsequently, precise carrier concentration optimization of the PbSe_0.6_S_0.4_Cu*_y_* system was carefully carried out by adjusting the Cu content, and the results are presented in Figure 3. Temperature-dependent electrical-transport properties for PbSe_0.6_S_0.4_Cu_*y*_ (*y* = 0.005, 0.014, 0.016, 0.018, 0.02) samples are illustrated in Figures 3(a)–3(c). As shown in Figures 3(a) and 3(b), both the temperature-dependent electrical resistivities and Seebeck coefficients show a “quasi” degenerate semiconductor behavior with a visible platform around 650 K, which is similar to the Cu-doped PbSe system [31]. This reveals clearly the dynamic doping effect in the PbSe_0.6_S_0.4_Cu_*y*_ system. Furthermore, a Cu-rich secondary phase is found to embed in the matrix (see SEM images in [Supplementary-material supplementary-material-1]), which is a prerequisite for Cu-doped lead chalcogenides to exhibit dynamic doping effect. Specifically, at 873 K, by varying the Cu content from 0.5 at% to 1.8 at%, the electrical resistivities substantially decrease from 30 × 10^−6^ *Ω*·m to 22 × 10^−6^ *Ω*·m, whereas the absolute values of the Seebeck coefficients decrease from 213 × 10^−6^ V·K^−1^ to 198 × 10^−6^ V·K^−1^. Further increased Cu content will lead to deterioration of electrical-transport properties, presumably due to a redundant Cu-rich secondary phase at high temperature, indicating that the solubility of the interstitial Cu at 873 K is close to 1.8 at%. Benefiting from the reinforced dynamic doping effect upon S alloying, the power factor reaches an outstanding value of 17 × 10^−4^ W·m^−1^·K^−2^ at 873 K for a 1.8 at% Cu-doped PbSe_0.6_S_0.4_ sample.

The temperature-dependent thermal-transport properties of Cu-doped PbSe_0.6_S_0.4_ samples are illustrated in Figures 3(d) and 3(e). As shown in Figure 3(d), the total thermal conductivities decrease with increasing temperature, which is a common phenomenon in heavily doped degenerate semiconductors. The lattice thermal conductivities have been calculated by subtracting *κ*_e_ from *κ*_tot_. *κ*_e_ is determined by the Wiedemann-Franz law, ,*κ*_*e*_ = *LσT* where *L* is the Lorenz number and *σ*is the electrical conductivity. It should be noted that *L* used in this work is calculated based on the single Kane band (SKB) model, which is lower than that calculated by the single parabolic band (SPB) model [36]. As shown in Figure 3(e), a 2 at% Cu-doped PbSe_0.6_S_0.4_ sample possesses the lowest thermal conductivity at room temperature, which is roughly consistent with our HTP screening result. It can also be observed from Figure 3(e) that the lattice thermal conductivities decrease continuously with the increasing Cu content, which is in agreement with our previous work [31, 32].

Benefiting from the modulated interstitial space, band gap, and density-of-state effective mass, the Cu-doped PbSe_0.6_S_0.4_ sample shows outstanding zT values at high temperature. As shown in Figure 3(f), a peak zT~1.6 at 873 K is achieved for the 1.8 at% Cu-doped PbSe_0.6_S_0.4_ sample, superior to most of the state-of-the-art *n*-type Te-free lead chalcogenides. Furthermore, to demonstrate the reliability of the HTP screening result, we have also evaluated the thermoelectric performance for low S alloying fraction samples. The results are presented in [Supplementary-material supplementary-material-1]. It is demonstrated that 2 at% Cu-doped PbSe_1‐*x*_S_*x*_ (*x* = 0.1, 0.2) samples show inferior peak zT values~1.2 and 1.3 at 873 K, which further confirms the validity and effectiveness of our HTP screening method.

## 3. Conclusions

In summary, we have achieved an ultrahigh thermoelectric performance in a Cu-doped PbSe-PbS system through a combined strategy including experimental HTP screening, theoretical transport property analysis, and further subtle property modulation. First, a satisfactory Se/S ratio for subsequently precise property modulation has been screened out by experimental HTP technique and transport model analysis. The theoretical consideration reveals that the demand for optimal carrier concentration of the PbSe-PbS system is noticeably increased due to the increase of *m*_d_^∗^, interstitial space, and band gap with increasing S alloying. Thus, the PbSe-PbS system may provide an ideal platform to achieve better dynamic doping effect, especially at the high temperature range. However, according to the transport model calculation, the alloying effect results in deteriorated overall thermoelectric performance. Therefore, in order to balance the two inversely correlated effects, the Se/S ratio is finally chosen to be 1.5 based on the experimental HTP screening results. Then, the thermoelectric performance has been further optimized by subtly adjusting the Cu content. As a result, an outstanding peak zT 1.6 at 873 K for a 1.8 at% Cu-doped (PbSe)_0.6_-(PbS)_0.4_ sample is achieved, which is a superior value among the *n*-type Te-free lead chalcogenides. It is worth noting that, for the HTP experimental screening of Se/S ratio, only two samples need to be presynthesized by the conventional method. This work demonstrates that the HTP experimental technique is very efficient and saves time to screen out the target compositions with high thermoelectric properties.

## 4. Materials and Methods

### 4.1. Sample Synthesis

High-purity Pb shots (>99.99%, Aladdin, China), Se chunks (>99.99% Aladdin, China), S powders (>99.9%), and Cu powder (>99.99%, Aladdin, China) were weighted and loaded into a silica tube according to the stoichiometric ratio of PbSe_1‐*x*_S_*x*_Cu_0.02_ (*x* = 0, 1) and PbSe_0.6_S_0.4_Cu_*y*_ (*y* = 0.005, 0.014, 0.106, 0.018). The silica tube was then sealed under vacuum. To ensure the *n*-type conduction for all samples, a small amount of excess Pb was intentionally added during the synthesis process. The silica tubes were then put in a computer-controlled furnace and slowly heated up to 1373 K at the heating rate of 1.5 K/min and then kept at this temperature for 11 h and followed by furnace cooling. The obtained ingots were ground into fine powders through an agate mortar and pestle. For the cylinder-like HTP sample with gradient compositions, it was densified by induction hot pressing in the dynamic vacuum at 823 K for 5 h under the uniaxial pressure of 50 MPa and then annealed at 673 K for 4 days. Samples for thermoelectric transport property measurement were also obtained by vacuum hot pressing under the uniaxial pressure of 70 MPa for 20 min; a high-density (>96% of theoretical density) pellet-like sample with a diameter of 10 mm was obtained for transport property measurements.

### 4.2. Sample Characterization

Micro area X-ray diffraction data for the HTP thin slab were collected by a Rigaku SmartLab-II diffractometer with Cu K_*α*_ radiation (40 kV × 30 mA). The SEM images and elemental mapping of the HTP thin slab and 2 at% Cu-doped PbSe_0.6_S_0.4_ sample were characterized by a scanning electron microscope (SEM, Gemini 300, Zeiss) equipped with an EDS detector (Oxford Instrument). The distribution of the Seebeck coefficient was scanned by a Potential-Seebeck Microprobe (PSM II, Panco Ltd., Germany). The thermal properties for the HTP thin slab was obtained by our home-made apparatus (see [Supplementary-material supplementary-material-1]). Room-temperature optical band gap was obtained by Fourier-transform infrared spectroscopy with the wavelength range from 2500 to 25000 nm (FTIR, VERTEX 70, Germany). The temperature-dependent electrical resistivity and Seebeck coefficient were characterized by the SEM-3 system (ULVAC-RIKO, Japan) under the protection of helium gas. The thermal conductivity was determined via *κ*_tot_ = *C*_p_ × *D* × *α*, where *C*_p_ is the heat capacity, *D* is the actual density of the sample, and *α* is the thermal diffusivity. The heat capacity was estimated *viaC*_p_ (*k*_B_/atom) = 3.07 + 0.00047 × (*T*‐300), which is commonly adopted to calculate heat capacity for lead chalcogenides. The density of the sample was determined by the Archimedes method, and the thermal diffusivity of the sample was measured by a laser flash apparatus (Netzsch LFA 457, Germany) with the Cowan model plus pulse correction.

## Supplementary Materials

Supplementary 1 Figure S1: micro area XRD patterns and derived lattice parameters for the HTP thin slab. Figure S2: images of home-made apparatus to characterize the thermal properties for an HTP thin slab. Figure S3: Pisarenko relation for PbSe_1‐*x*_S_*x*_ (*x* = 0, 0.1 0.2, 0.3, 0.4, 0.5, 0.6, 0.7, 0.8, 0.9, 1) at 300 K and 850 K. Figure S4: room-temperature XRD patterns and FTIR spectra for PbSe_1‐*x*_S_*x*_ (*x* = 0, 0.1, 0.3, 0.7) samples. Figure S5: SEM images for the 2 at% Cu-doped PbSe_0.6_S_0.4_ sample. Figure S6: temperature dependency of thermoelectric transport properties for 2 at% Cu-doped PbSe_1‐*x*_S_*x*_ (*x* = 0.1, 0.2) samples. Table S1: lattice parameters of the corresponding micro regions of the HTP sample. Table S2: room-temperature carrier concentration and mobility for (1‐*x*)(PbCu_0.02_Se):*x*(PbCu_0.02_S) (*x* = 0.1, 0.2, ⋯, 0.6) samples. Table S3: physical parameters of PbSe and PbS for modeling and the details of the calculation by the SKB model and the Klemens model and the expression of the thermoelectric quality factor.

Supplementary 2 Movie S1: introduction to the automatic batching and prepressing system.
