# Supplementary material for: High Thermoelectric Performance of Cu-Doped PbSe-PbS System Enabled by High-Throughput Experimental Screening
Source: Research (Wash D C). 2020 Mar 7;2020:1736798. doi: 10.34133/2020/1736798 (PMC7080995; doi:10.34133/2020/1736798)
Supplement: Supplementary 1 — Figure S1: micro area XRD patterns and derived lattice parameters for the HTP thin slab. Figure S2: images of home-made apparatus to characterize the thermal properties for an HTP thin slab. Figure S3: Pisarenko relation for PbSe1‐xSx (x = 0, 0.1 0.2, 0.3, 0.4, 0.5, 0.6, 0.7, 0.8, 0.9, 1) at 300 K and 850 K. Figure S4: room-temperature XRD patterns and FTIR spectra for PbSe1‐xSx (x = 0, 0.1, 0.3, 0.7) samples. Figure S5: SEM images for the 2 at% Cu-doped PbSe0.6S0.4 sample. Figure S6: temperature dependency of thermoelectric transport properties for 2 at% Cu-doped PbSe1‐xSx (x = 0.1, 0.2) samples. Table S1: lattice parameters of the corresponding micro regions of the HTP sample. Table S2: room-temperature carrier concentration and mobility for (1‐x)(PbCu0.02Se):x(PbCu0.02S) (x = 0.1, 0.2, ⋯, 0.6) samples. Table S3: physical parameters of PbSe and PbS for modeling and the details of the calculation by the SKB model and the Klemens model and the expression of the thermoelectric quality factor. [file 1736798.f1.docx]

Supplementary Materials

# High Thermoelectric Performance of Cu-doped PbSe-PbS System Enabled by High-throughput Experimental Screening and Precise Property Modulation

Li You^1^, Zhili Li^1^, Quanying Ma^1^, Shiyang He^1^, Qidong Zhang^1^, Feng Wang^1^, Guoqiang Wu^1^, Qingyi Li^1^, Pengfei Luo^1^, Jiye Zhang^1^*, and Jun Luo^1,2^*

^1^ School of Materials Science and Engineering, Shanghai University, 99 Shangda Road, Shanghai 200444,

*^2^Materials Genome Institute, Shanghai University, 99 Shangda Road, Shanghai 200444, China.*

Correspondence should be addressed to Jun Luo; junluo@shu.edu.cn and Jiye Zhang; jychang@shu.edu.cn

**1. Supplementary Figures**


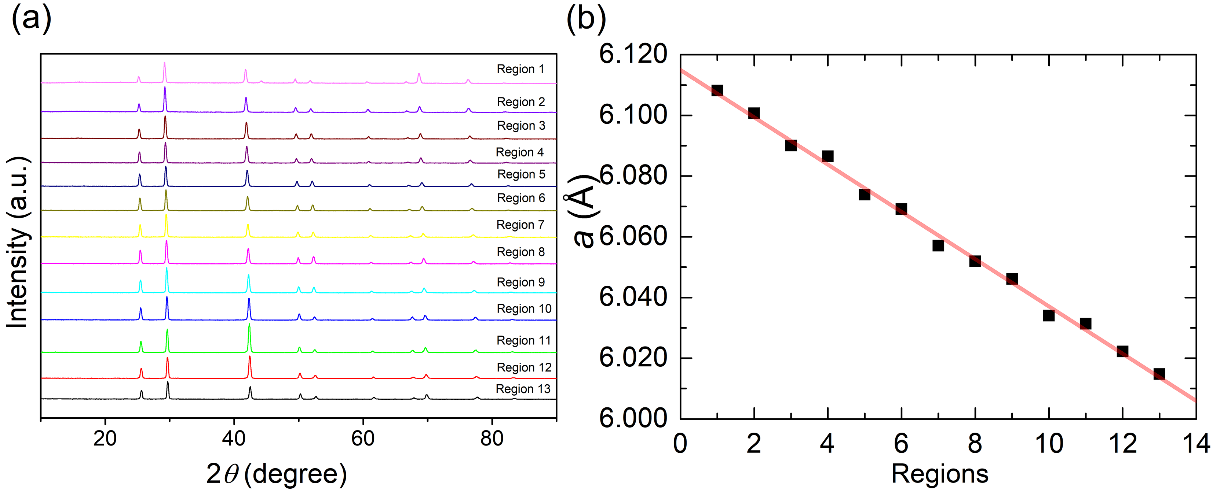


Figure S1. (a) Micro area XRD patterns of serial regions for HTP thin slab shown in Figure 1 in the main text; (b) Lattice parameters derived from the XRD data of correlated regions presented in (a).


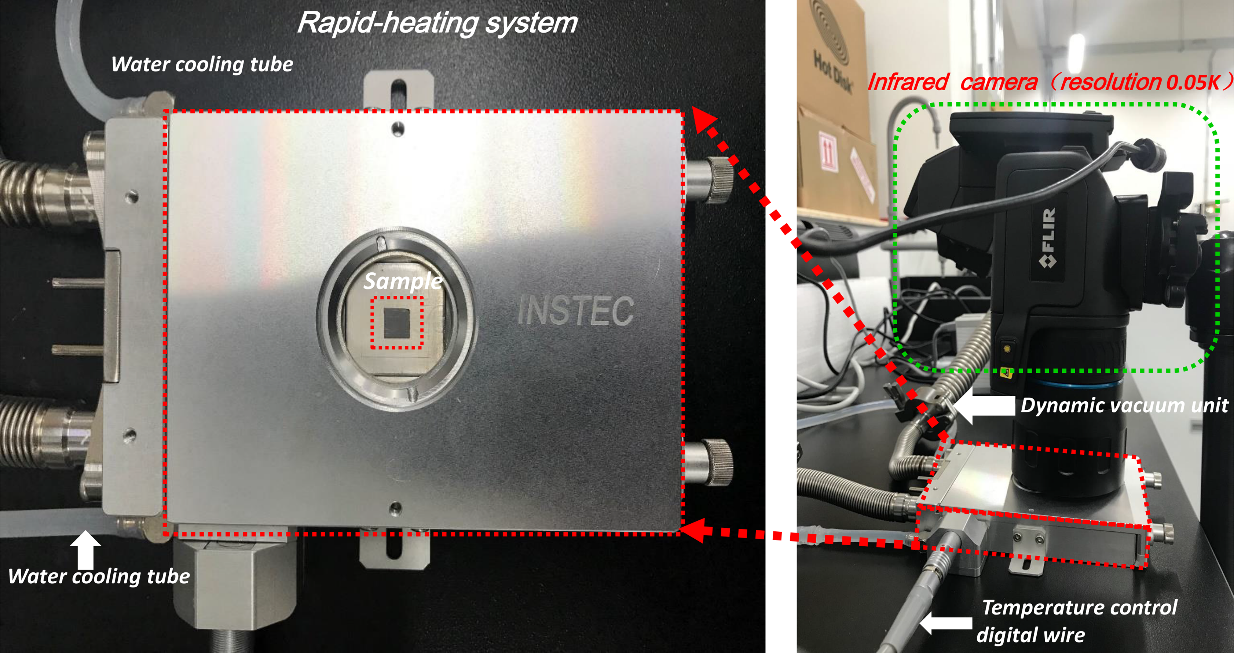


Figure S2. Home-made apparatus for thermal transport property screening of the HTP thin slab.


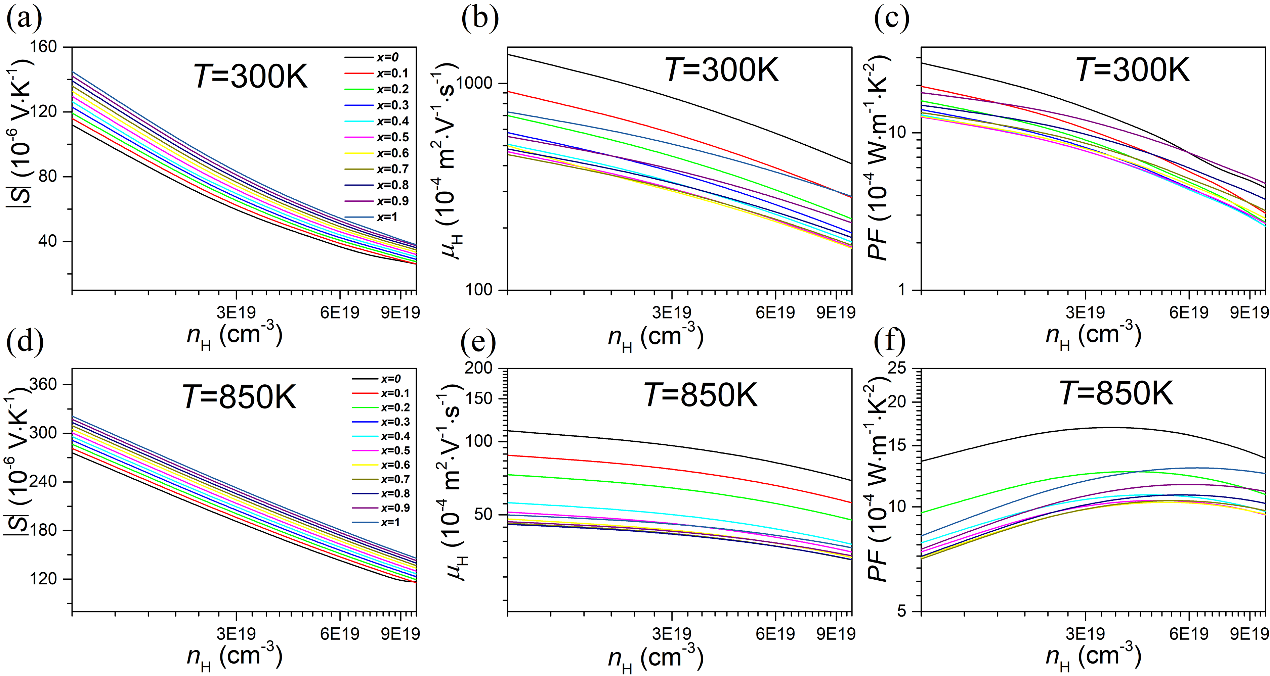


Figure S3. Pisarenko relation for PbSe_1-_*_x_*S*_x_* (*x*=0, 0.1 0.2, 0.3, 0.4, 0.5, 0.6, 0.7, 0.8, 0.9, 1) at 300K and 850K. Carrier concentration dependent (a) and (d): Seebeck coefficient (absolute value), (b) and (e): Hall mobility, (c) and (f): power factor. All the solid lines presented in Figure S3 are predicted by SKB model with the assumption of acoustic phonon and alloying scattering dominate electron transport.


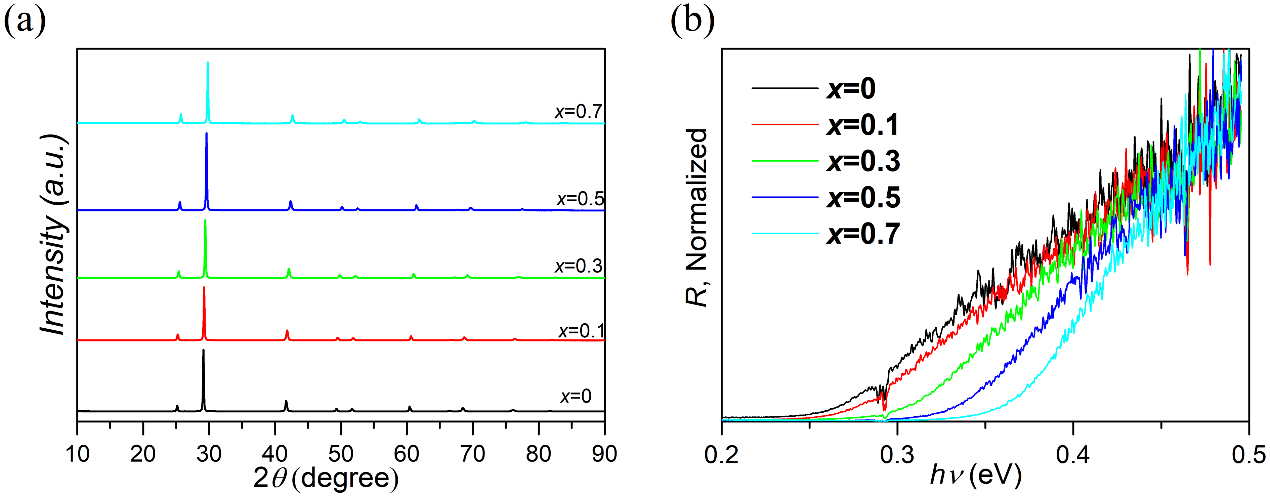


Figure S4. (a) XRD patterns and (b) FTIR spectra of undoped PbSe_1-_*_x_*S*_x_* (*x*=0, 0.1, 0.3, 0.5, 0.7) samples, which were synthesized to verify our assumption of the enlarged band gap by S alloying.


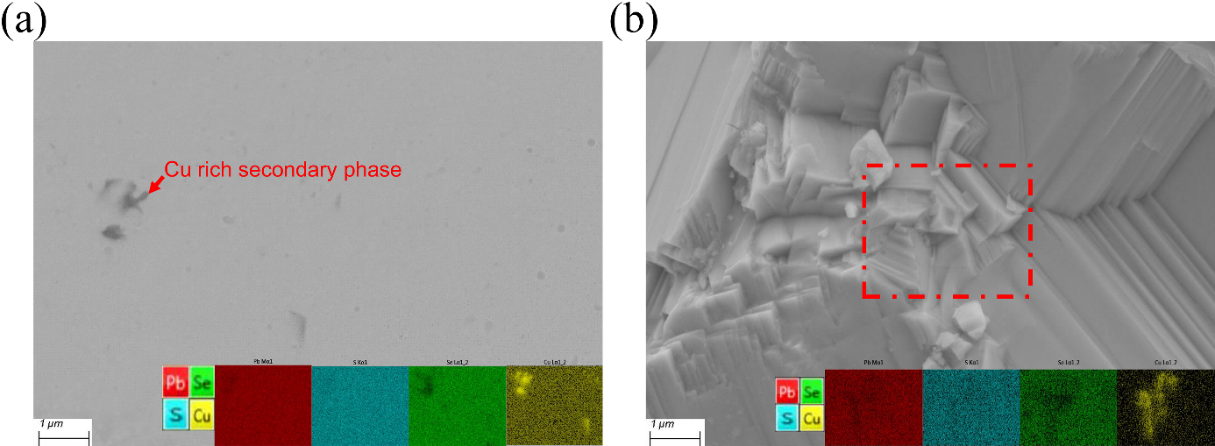


Figure S5. SEM images for 2 at% Cu doped PbSe_0.6_S_0.4_ sample. (a) Secondary electron (SE) images of the polished surface and corresponding energy dispersive spectroscopy (EDS) elemental mappings for all elements. The red arrow in (a) indicates the Cu-rich secondary phase; (b) SE images for [fracture surface](javascript:;). EDS mapping results presented in (b) are taken from red-dashed rectangle area. The EDS mapping results reveal that the Cu element is enriched at grain boundaries, which is a prerequisite condition for Cu dynamic doping effects.

**
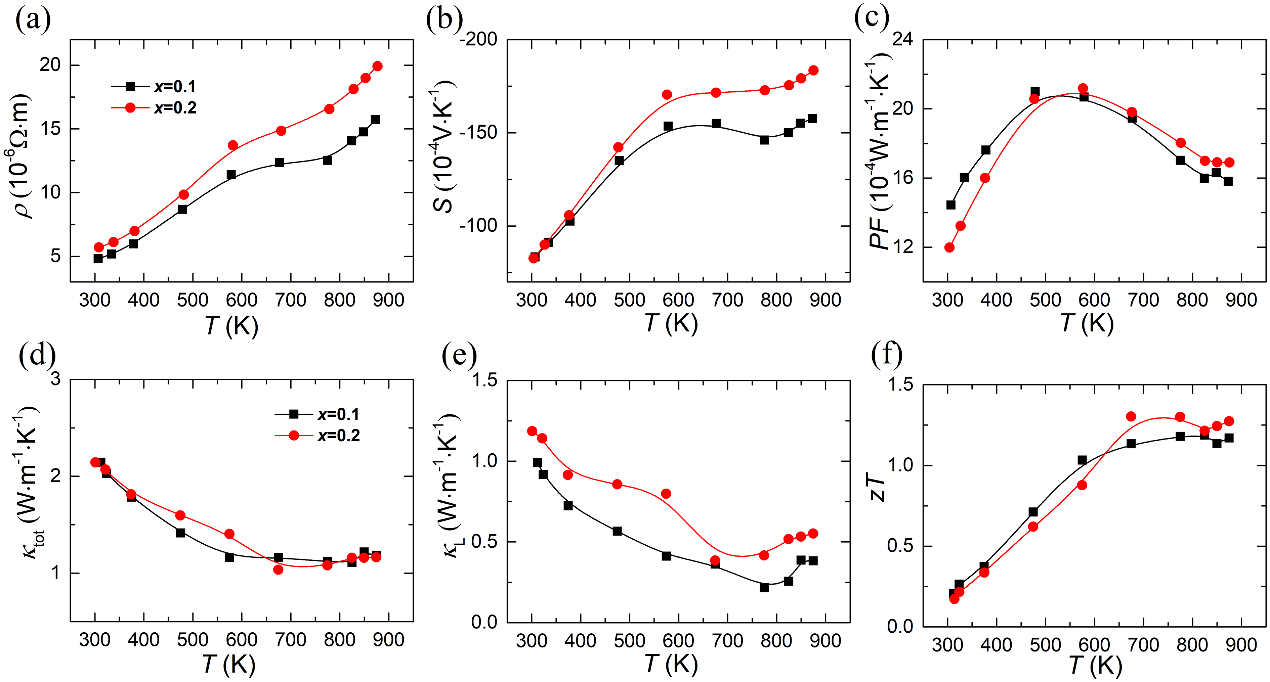
**

Figure S6. Temperature-dependent thermoelectric transport properties for 2 at% Cu doped PbSe_1-_*_x_*S*_x_* (*x*=0.1, 0.2) samples. (a) Electrical resistivities, (b) seebeck coefficients, (c) power factors, (d) total thermal conductivities, (e) lattice thermal conductivities and (f) figure of merit *zT*.

**2. Supplementary Tables**

Table S1. Lattice parameters of the corresponding micro regions of the HTP sample.

| Mciro Region | *a* (Å) |  | Mciro Region | *a* (Å) |
| --- | --- | --- | --- | --- |
| 1 | 6.1081(5) |  | 8 | 6.0519(5) |
| 2 | 6.1007(9) |  | 9 | 6.0461(7) |
| 3 | 6.0901(6) |  | 10 | 6.0340(7) |
| 4 | 6.0865(5) |  | 11 | 6.0314(4) |
| 5 | 6.0739(8) |  | 12 | 6.0222(6) |
| 6 | 6.0691(5) |  | 13 | 6.0148(5) |
| 7 | 6.0571(5) |  |  |  |

Table S2. Room temperature Hall carrier concentration and mobility for (1-*x*)(PbCu_0.02_Se):*x*(PbCu_0.02_S) (*x*=0.1, 0.2, ...0.6) samples.

| Compositions | *n*_H_ (cm^-3^) | *μ*_H_(cm^2^﹒V^-1^﹒s^-1^) |
| --- | --- | --- |
| PbSe_0.9_S_0.1_-2 at% Cu | 1.6×10^19^ | 621 |
| PbSe_0.8_S_0.2_-2 at% Cu  PbSe_0.7_S_0.3_-2 at% Cu  PbSe_0.6_S_0.4_-2 at% Cu  PbSe_0.5_S_0.5_-2 at% Cu  PbSe_0.4_S_0.6_-2 at% Cu | 1.8×10^19^  2.0×10^19^  1.5×10^19^  1.6×10^19^  1.6×10^19^ | 454  460  418  241  262 |

Table S3. Physical parameters of PbSe and PbS used for modeling in this work.

| Parameter | PbSe | PbS |
| --- | --- | --- |
| *m*_d_^*^ at 300K for conduction band (*m*_e_)  Energy band gap at L point *E*_g_ (eV) | 0.27  0.29 | 0.39  0.42 |
| *b*, *m*_d_^*^~*T*^b^ (L band) at *T*＜800K | 0.5 | 0.4 |
| Deformation potential coefficient$\Xi$(eV) | 25 | 27 |
| Inertial effective mass *m*_i_^*^ at 300K (eV)  Band degeneracy (L band) *N*_V_  Longitudinal elastic moduli *C*_l_ (×10^-10^ Pa)  Band anisotropy factor (Conduction band) *K*  Lattice parameter *a* (Å)  Molar mass (g/mol)  Grüneisen constant $\gamma$ Average sound velocity *V*_ave_ (m/s)  Debye temperature *θ*_D_ (K)  *κ*_L_ at 300K (W﹒m^-1^﹒K^-1^)  *κ*_L_ at 850K (W﹒m^-1^﹒K^-1^) | 0.11  4  9.1  1.75  6.13  286.2  1.65  3220  190  1.6  0.7 | 0.15  4  11.1  1.3  5.49  239.3  2  3460  300  2.5  1 |

**3. Theoretical transport models for PbSe-PbS solid solutions**

**3.1 SKB model**

The electrical transport properties of n-type PbSe-PbS solid solutions can be modeled by adopting the single Kane band model (SKB) by assuming that acoustic phonon scattering and alloy scattering dominate the electron transport. It is to note that in the current model, carrier scattering from optical phonons optical phonons through polar scattering is not taken into consideration. Therefore, the Hall mobility for high S content might be slightly overestimated, especially at low carrier concentration range (1~7×10^18^ cm^-3^). However, for a heavily doped n-type PbSe-PbS system, if the carrier concentration is sufficiently high (above 1×10^19^ cm^-3^), the contribution of polar scattering from optical phonons on electron transport could be negligible. Besides, the polar scattering will be weakened and the acoustic phonon will dominate the charge transport at elevated temperature. Therefore, neglecting the effect of the polar scattering is a reasonable approximation in modeling the electrical-transport properties [1]. For the SKB model, the thermoelectric-transport parameters can be expressed as follows: [1, 2]

Seebeck coefficient: $S=\frac{k_{B}}{e}\left(\frac{\int_{0}^{\infty} \left(-\frac{\partial f}{\partial\varepsilon}\right)\tau_{total}(\varepsilon)\varepsilon^{5/2}(1+\varepsilon\alpha)^{3/2}(1+2\varepsilon\alpha)^{-1}d\varepsilon}{\int_{0}^{\infty} \left(-\frac{\partial f}{\partial\varepsilon}\right)\tau_{total}(\varepsilon)\varepsilon^{3/2}(1+\varepsilon\alpha)^{3/2}(1+\varepsilon\alpha)^{-1}d\varepsilon}-\eta\right)$

Carrier concentration：

Carrier mobility: $\mu=\frac{e}{m_{I}^{*}}\frac{\int_{0}^{\infty} \left(-\frac{\partial f}{\partial e}\right)\tau_{total}(\varepsilon)(\varepsilon+\alpha\varepsilon^{2})^{3/2}(1+2\alpha\varepsilon)^{-1}d\varepsilon}{\int_{0}^{\infty} \left(-\frac{\partial f}{\partial\varepsilon}\right)(\varepsilon+\alpha\varepsilon^{2})^{3/2}d\varepsilon}$

Hall factor *A* ($n=A\times n_{\text{H}}$):$A=\frac{3K(K+2)}{(2K+1)^{2}}\frac{\int_{0}^{\infty} \left(-\frac{\partial f}{\partial\varepsilon}\right)\tau_{total}(\varepsilon)^{2}\varepsilon^{3/2}(1+\varepsilon\alpha)^{3/2}(1+2\varepsilon\alpha)^{-2}d\varepsilon\int_{0}^{\infty} (-\frac{\partial f}{\partial\varepsilon})\varepsilon^{3/2}(1+\varepsilon\alpha)^{3/2}d\varepsilon}{\left(\int_{0}^{\infty} \left(-\frac{\partial f}{\partial\varepsilon}\right)\tau_{total}(\varepsilon)\varepsilon^{3/2}(1+\varepsilon\alpha)^{3/2}(1+2\varepsilon\alpha)^{-1}d\varepsilon\right)^{2}}$Lorenz number:

$L=\left(\frac{k_{B}}{e}\right)^{2}\left[\frac{\int_{0}^{\infty} \left(-\frac{\partial f}{\partial\varepsilon}\right)\tau_{total}(\varepsilon)\varepsilon^{7/2}(1+\varepsilon\alpha)^{3/2}(1+2\varepsilon\alpha)^{-1}d\varepsilon}{\int_{0}^{\infty} \left(-\frac{\partial f}{\partial\varepsilon}\right)\tau_{total}(\varepsilon)\varepsilon^{3/2}(1+\varepsilon\alpha)^{3/2}(1+\varepsilon\alpha)^{-1}d\varepsilon}-\left(\frac{\int_{0}^{\infty} \left(-\frac{\partial f}{\partial\varepsilon}\right)\tau_{total}\left(\varepsilon\right)\varepsilon^{5/2}\left(1+\varepsilon\alpha\right)^{3/2}\left(1+2\varepsilon\alpha\right)^{-1}d\varepsilon}{\int_{0}^{\infty} \left(-\frac{\partial f}{\partial\varepsilon}\right)\tau_{total}\left(\varepsilon\right)\varepsilon^{3/2}\left(1+\varepsilon\alpha\right)^{3/2}\left(1+2\varepsilon\alpha\right)^{-1}d\varepsilon}\right)^{2}\right]$Relaxation time by acoustic phonon scattering:

Relaxation time by alloy scattering:

The total relaxation time can be obtained by Matthiessen’s rule: $\tau_{total}^{-1}=\tau_{ac}^{-1}+\tau_{alloy}^{-1}$.

It should be noted that the band nonparabolicity is taken into account in the calculations of the relaxation time by both acoustic phonon scattering and alloy scattering.

In the above equations, *η* is the reduced chemical potential of charge carriers in the system*. f* represents the Fermi distribution function. *m*_d_*^*^* is the density-of-state effective mass for the condction band edge. *m_I_^*^* is the inertial mass, which can be expressed as *m_I_^*^=3(1/m*_∥_^*^+2/ *m*_⊥_^*^*)*. *m_b_*^*^ is the band mass that can be calculated *via m_b_*^*^=*N_V_* ^-2/3^*m*_d_*^*^*= (*m*_∥_^*^ *m*_⊥_^*^)*^1/3^*. *N_V_* is the band degeneracy, *ɛ* is the reduced energy of the electron state, and *α* (*α*=*k_B_T*/*E_g_*) is the reciprocal reduced band separation responsible for the nonparabolicity of the band. *Ω* is the volume per atom. *U* is the alloying scattering potential, which determines the magnitude of the alloy scattering for the given alloy. *E_g_* is the direct band gap at the *L* point of the Brillouin zone. *K* is the band anisotropy factor defined as *K= m*_∥_*/*m*_⊥_^*^. *C_l_* is the longitudinal elastic moduli, and *Ξ* is the deformation potential. The transport coefficents of PbSe-PbS system used in this work are taken from the linear average between two binary compounds. For PbSe and PbS, the aformentioned transport parameters are listed in Table S1.

**3.2 Klemens model**

The thermal-transport properties of PbSe-PbS solid solutions can be modeled by adopting the Klemens model. This model is valid when the temperature is above Debye temperature, where the influence of grain boundary scattering on the *κ*_L_ is negligible. Besides, this model takes only the Umklapp and point defect phonon scattering into consideration. The ratio *κ*_L,alloy_ of the alloyed crystal to that without disorder, *κ*_L,pure_, can be expressed as: [2]

*Ω* is the volume per atom, *v* is the sound velocity, *θ*_D_ is the Debye temperature, *Γ* is the scattering parameter, which usually consists two parts: mass difference and strain field difference. For A_1-_*_x_*B*_x_* type or pseudo-binary (AB)_1-_*_x_*(AC)*_x_* type compound, the *Γ* can be expressed as: $\Gamma=x\left(1-x\right)\left[\left(\frac{\Delta M}{M}\right)^{2}+\varepsilon\left(\frac{\Delta a}{a}\right)^{2}\right]$, where Δ*M* and Δ*α*are the mass and lattice constant difference between two constituents [2]. *ε* is the phenomenological parameter that can be calculated *via* $\varepsilon=\frac{2}{9}\left[\left(G+6.4\gamma\right)\frac{1+r}{1-r}\right]^{2}$. In the above equations, *γ* is the Grüneisen parameter, *r* is the Poisson ratio. G is the ratio between the contrasts in bulk modulus and that in the local bonding length. For lead chalcogenides, *G*=3 and calculated *ε* for PbSe and PbS are 110 and 150, respectively. All the parameters used for modeling are taken form the linear average of two binary compounds, which are listed in Table S1.

**3.3 Thermoelectric quality factor**

By combining the calculated Hall mobility, lattice thermal conductivity and *m*_d_^*^, the material’s quality factor *β* can be obtained theoretically, which can be defined by the following equation: [1]

In the above equation, *m*_e_ is the free electron mass, *μ*_w_ is the weight mobility that can be calculated *via μ*_w_=*μ*_0_×(*m*_d_^*^/m_e_)^1.5^, where *μ*_0_ is the degenerate limit for the undoped sample.

**References**

[1] K. Koumoto and T. Mori, *Thermoelectric nanomaterials: materials design and applications.* Springer, 2013.

[2] H. Wang, A. D. LaLonde, Y. Z. Pei and G. J. Snyder, *Adv. Funct. Mater.*, 2013, **23**, 1586-1596.
